# On the Mechanism of Human Red Blood Cell Longevity: Roles of Calcium, the Sodium Pump, PIEZO1, and Gardos Channels

**DOI:** 10.3389/fphys.2017.00977

**Published:** 2017-12-12

**Authors:** Virgilio L. Lew, Teresa Tiffert

**Affiliations:** Physiological Laboratory, Department of Physiology, Development and Neuroscience, University of Cambridge, Cambridge, United Kingdom

**Keywords:** human red blood cell, programmed senescence, calcium homeostasis, sodium pump, PIEZO1, Gardos channel, xerocytosis, erythrocyte longevity

## Abstract

In a healthy adult, the transport of O_2_ and CO_2_ between lungs and tissues is performed by about 2 · 10^13^ red blood cells, of which around 1.7 · 10^11^ are renewed every day, a turnover resulting from an average circulatory lifespan of about 120 days. Cellular lifespan is the result of an evolutionary balance between the energy costs of maintaining cells in a fit functional state versus cell renewal. In this Review we examine how the set of passive and active membrane transporters of the mature red blood cells interact to maximize their circulatory longevity thus minimizing costs on expensive cell turnover. Red blood cell deformability is critical for optimal rheology and gas exchange functionality during capillary flow, best fulfilled when the volume of each human red blood cell is kept at a fraction of about 0.55–0.60 of the maximal spherical volume allowed by its membrane area, the optimal-volume-ratio range. The extent to which red blood cell volumes can be preserved within or near these narrow optimal-volume-ratio margins determines the potential for circulatory longevity. We show that the low cation permeability of red blood cells allows volume stability to be achieved with extraordinary cost-efficiency, favouring cell longevity over cell turnover. We suggest a mechanism by which the interplay of a declining sodium pump and two passive membrane transporters, the mechanosensitive PIEZO1 channel, a candidate mediator of P_sickle_ in sickle cells, and the Ca^2+^-sensitive, K^+^-selective Gardos channel, can implement red blood cell volume stability around the optimal-volume-ratio range, as required for extended circulatory longevity.

## Introduction

Human red blood cells (RBCs) have a prescribed lifespan of about 4 months before being cleared from the circulation. During this period, RBCs devoid of organelles and biosynthetic capacity experience irreversible age-related changes in metabolism, membrane transport, ionic composition, cortical cytoskeleton, and immune-reactivity, among others (Beutler, 1985b; Clark, 1988; Romero and Romero, 1999; Lew et al., 2007; Tiffert et al., 2007; Lutz, 2012; Franco et al., 2013; Lutz and Bogdanova, 2013). Nevertheless, under the microscope, the appearance of RBCs from healthy adults remains remarkably uniform, regardless of cell age. In narrow age-cohorts, the coefficient of variation in the haemoglobin concentration was found to be only about 3% (Lew et al., 1995). Such uniformity can only result from an erythroid production sequence that closely coordinates membrane area, volume, osmolyte, and haemoglobin contents in each cell, so that cells with larger areas also have larger volumes and haemoglobin contents, and vice versa. The mature RBC product is equipped with components capable of maintaining an extraordinary level of homogeneity in discoid appearance and haemoglobin concentration throughout a long circulatory lifespan (Svetina, 1982; Lew et al., 1995). That such manufacturing precision can be maintained by a bone marrow producing about 7 · 10^9^ cells per hour in a normal adult is a truly remarkable feat of evolutionary bioautomation. And no less remarkable is the mechanism that enables the homeostatic stability of the RBCs throughout senescence. In this review we argue that maintenance of RBC longevity is driven primarily by the need to compensate for unavoidable reductions in sodium pump activity, which, if unchecked, would lead to cell swelling and early loss of the deformability required for normal capillary flow. We suggest that the need to maintain optimal circulatory performance has driven the evolution of a remarkably cost-efficient compensation strategy to preserve RBC volume and to extend RBC lifespan.

## On the importance of maintaining RBC discoid shape

Before considering *how* shape is maintained despite so much age-related change, let us try to answer *why*. Because selective pressures guide adaptive change to optimize function the answer must lie with the basic RBC function of mediating gas transfer between lungs and tissues. Gas exchange is a passive diffusional process that poses no direct metabolic demand, but requires a rheologically competent cell (Kaestner and Bogdanova, 2014). The discocyte shape allows RBCs to deform, fold, and squeeze against the endothelial walls of capillaries, exposing maximal surface area thus offering minimal diffusional distances for rapid O_2_ and CO_2_ exchanges across the capillary walls. Thus, maintenance of the discocyte shape is essential for preserving the optimal viability and functional capacity of the cells for an extended circulatory lifespan. In general, the basic requirement for optimal RBC rheology is maintenance of the cell volume substantially below the maximal spherical volume that can be accommodated by the membrane area of each cell. As stressed by Pivkin et al. (2016), the surface to volume ratio is by far the most important parameter of RBC deformability. In normal healthy human RBCs with favourable surface-volume ratios, rheology optimization is fulfilled by a discoid shape resulting from the biophysical properties of its membrane. In RBCs from other species the same optimization principles are fulfilled by a variety of other shapes, with different underlying cytoskeletal structures and biophysical properties (Cossins and Gibson, 1997).

RBC volumes are kept within 55–60% of their maximal spherical volumes. Let us call this the optimal volume ratio, or OVR. OVR is better known by its inverse, the critical haemolytic volume, which has values around 1.7 times the normal RBC volume (Ponder, 1948, 1950; Lew et al., 1995). With OVR values above 65% (swelling), and below 50% (dehydration), cell deformability and rheology become compromised, though by different mechanisms at each end (Secomb, 1987; Secomb and Hsu, 1995; Derganc et al., 2003; Fedosov et al., 2014a,b; Gompper and Fedosov, 2016; Lanotte et al., 2016). In this light, the discoid shape is simply the observable representation of OVR. Keeping RBC volumes within the physiological OVR range over such an extended circulatory longevity requires an extraordinary level of volume control, and this is where metabolic energy is invested.

## Key features of volume control in human RBCs

Volume control in mature RBCs is entirely dependent on the combined function of a set of active and passive native membrane transporters (Garrahan and Glynn, 1967; Garrahan and Garay, 1974; Schatzmann, 1983; Rega and Garrahan, 1986; Carafoli, 1992). Maintenance of cellular homeostasis over extended periods of time, relying only on an assembly of native transporters unable to be repaired or replaced, represents an amazing evolutionary gamble. While ensuring cell stability under normal conditions, in inherited haematological disorders such as sickle cell disease (Lew and Bookchin, 2005), thalassaemia (Weatherall, 1997, 2004), or hereditary xerocytosis (Houston et al., 2011; Zarychanski et al., 2012; Andolfo et al., 2015; Alper, 2017; Fermo et al., 2017; Glogowska et al., 2017), altered membrane transport becomes a serious liability. In the following sections, the terms “permeability” and “leak” will be used to represent subsets of passive membrane transporters mediating the fluxes of the indicated substrates.

The volume stability of the highly water-permeable RBC depends on two key properties, a low permeability to cations, and a high permeability to neutral solutes such as glucose or urea. The low cation permeability severely rate-limits net salt and isoosmotic fluid transfer (Lew and Beaugé, 1979). The high neutral solute permeability allows rapid solute equilibration with minimal osmotic stress and volume displacement.

The most important evolutionary advantage associated with a low cation permeability is to allow volume control with negligible cost to RBC glycolytic metabolism and to the organism as a whole. The RBC ion leaks are balanced by two ATP-fuelled pumps, the sodium pump (ATP1) and the plasma membrane calcium pump (PMCA). The pump-leak turnover rate of Na^+^ and K^+^, of about 2–3 mmol/(L cells.h) in middle-age RBCs, representing mean population values, is sufficient to keep the Na^+^ and K^+^ concentration balance of the cells, and to offset the colloidosmotic swelling force of haemoglobin with minimal demand on glycolytic ATP production. The physiological pump-leak turnover rate of calcium in physiological conditions is about 20–50 μmol/(L cells.h; Lew et al., 1982), a rate three orders of magnitude below the Ca^2+^ extrusion capacity of a Ca^2+^-saturated PMCA (Dagher and Lew, 1988; Lew et al., 2003). Thus, overall ATP turnover rates of about 1.0–2.0 mmol/(L cells.h) cover all the metabolic demands of normal, mature, middle-aged RBCs. In a healthy adult with a total red cell volume of ~2 L and a total body ATP turnover of ~5 mol/h, RBC ATP turnover contributes a negligible 0.04–0.06% to the total. This makes the low cation permeability of RBCs a most economical and efficient determinant of volume stability and preserved functional competence, enabling the evolution of a gas-carrier cell devoid of encumbering energy producing, and consuming structures and with a long circulatory lifespan. This is a general strategy, followed in many species, with substantial variations in the nature of the membrane transporters involved (Parker, 1973, 1978).

## A failing sodium pump presents the major challenge to RBC longevity

An extended RBC lifespan is a major saver in expensive cell manufacture and turnover, and hence a powerful selective drive for cell preservation over replacement (Beutler, 1985a,b, 1986). In this light, the best recipe for extended RBC longevity would be maintenance of a constant volume and density within the OVR range. However, lack of biosynthetic capacity and protein renewal makes RBC volume stability vulnerable to cumulative damage of the enzymes and transporters involved in its control. Despite the robust antioxidant machinery of RBCs (Arese et al., 2005; Lutz, 2012; Lutz and Bogdanova, 2013), cumulative effects of non-enzymic oxidation, glycation, and of other processes, unavoidably alter protein structure and function (Beutler, 1985a,b, 1986; Gonzalez Flecha et al., 1999; Raftos et al., 2001; Keller et al., 2015; Piedrafita et al., 2015). Let us consider first the specific age-related changes relevant to volume control, and then the nature of the adaptive changes critical for extending the flow viability of aging RBCs.

Of the many documented changes observed in aging RBCs, those most threatening to volume stability are the gradual reduction in the activity of the sodium pump, and the progressive decline in glycolysis and ATP levels. From the vast body of experimental work in many different laboratories over the last six decades, the following essential facts emerge with uncontroversial consistency: (i) the number of sodium pumps per cell, estimated by ^3^H-Ouabain binding, declines with RBC age by up to 70%; (ii) in aging RBCs the trans-membrane gradients of Na^+^ and K^+^ decline steadily, with increased Na^+^ and decreased K^+^ contents, all within relatively small variations in total [Na^+^] + [K^+^]; and (iii), ATP levels fall by up to 40% in aging RBCs (Cohen et al., 1976, 2008; Joiner and Lauf, 1978; Magnani et al., 1983; Cheng et al., 1984; Clark, 1988; Lew et al., 2007; Franco et al., 2013; Lutz and Bogdanova, 2013).

Dramatic changes were also documented in the activity of other transport-mediated processes, in particular the reductions in calcium pump and Gardos channels activity. However, Ca^2+^ extrusion and Gardos channel-mediated K^+^ transport are hardly affected because of the huge native spare capacities of these transporters (Garcia-Sancho and Lew, 1988; Romero and Romero, 1997, 1999; Lew et al., 2003, 2007; Tiffert et al., 2007). Thus, the main focus for the need of volume control remains with the Na pump.

Decline in Na pump activity on its own would lead to a slow dissipation of the sodium and potassium gradients with a net gain of NaCl in excess of KCl loss, cell swelling and density decrease. The important issue to note here is that when RBCs start circulatory life, transiting from reticulocyte to mature RBC, their volume is at or just above the upper limit of the OVR (Rapoport, 1986). Therefore, any uncompensated decline in sodium pump activity, whatever its rate, would lead to increases in RBC volume, placing the volume ratio further above its optimal range and increasingly compromising rheology and gas exchange functionality with time, more so the higher the rate of pump inactivation. Thus, balancing the inevitable decline in sodium pump activity in order to keep the cells within safe OVR ranges would seem to offer the best conditions for RBC longevity to evolve.

## The age-density pattern

Instead of swelling, as predicted by uncompensated Na pump decline, mature RBCs shrink gradually with age. This was ascertained long ago by following the distribution of ^59^Fe-labelled heme in layers of density-fractionated RBCs after a single intravenous injection of the isotope (Borun et al., 1957; Beutler, 1986; Clark, 1988). These experiments documented the senescence pattern of human RBCs, determined its duration, and established density separation as the method of choice to explore age-related changes in human RBC properties. However, a minor twist in this story has been systematically overlooked. When the ^59^Fe activity ratios between top and bottom cell-density layers were monitored for up to 200 days following tracer injections, the ratios were seen to decline steadily, at variable rates in different subjects, up to day 70. But then, between days 70 and 120 the ratios changed direction suggesting a terminal density reversal (Borun et al., 1957). A minor part of this density reversal could be attributed to reutilization of diluted tracer, as discussed by Clark (1988), but most of it reflects a genuine and gradual reduction in the density of RBCs before their immune clearance from the circulation (Kay, 1975; Lutz et al., 1987; Lutz and Bogdanova, 2013), a view also supported by recent additional evidence (Bookchin et al., 2000; Franco et al., 2013; Lew and Tiffert, 2013). Explanation of this age-density pattern requires consideration of the transport processes involved in the control of RBC homeostasis.

## Mechanisms of age-related RBC densification and terminal reversal

Two passive transporters of human RBCs deserve special attention: PIEZO1, a mechanosensitive ion channel (Zarychanski et al., 2012; Andolfo et al., 2013; Shmukler et al., 2014; Cinar et al., 2015; Kaestner, 2015; Alper, 2017; Glogowska et al., 2017), and the Ca^2+^-sensitive, K^+^-selective Gardos channel (KCNN4) (Gardos, 1958; Lew and Ferreira, 1978; Ghanshani et al., 1998; Hoffman et al., 2003; Gottlieb et al., 2012; Gnanasambandam et al., 2015; Fermo et al., 2017; Rivera et al., 2017). PIEZO1 mutations were found to be responsible for marked RBC dehydration in hereditary xerocytosis (HX), a clinically heterogeneous family of congenital haemolytic anaemias. Detailed studies revealed that mutant channels exhibited a number of kinetic abnormalities relative to wild-type channels of which the most prominent was a marginally reduced inactivation kinetics following brief stretch-activation pulses (Bae et al., 2013). If a relatively small inactivation delay can lead to such a profound dehydration in RBCs from HX subjects (Glogowska et al., 2017), the wild-type channel may be expected to contribute to the progressive dehydration of normal RBCs over periods of weeks in the circulation. Moreover, in mice with specific deletion of PIEZO1 from the haematopietic system the RBCs were found to become over-hydrated just as expected from the removal of a transport pathway involved in a volume-balancing dehydration chain (Cahalan et al., 2015).

In addition, whole-cell patch-clamp recordings of normal human RBCs showed that brief suction pulses through the patch pipette elicited a Ca^2+^ influx sufficient to activate secondary currents through Gardos channels (Dyrda et al., 2010), well within the response expected from a brief and sharp increase in Ca^2+^ permeability through stretch-activated PIEZO1 channels in the microcirculation. This experimental condition is reminiscent of the one generated in sickled cells, where the same topology of membrane deformation is generated from inside the cells by protruding polymers of deoxy-haemoglobin S. In sickle cells, consecutive deoxygenation episodes triggered reversible increases in the calcium permeability of all the RBCs, but the dehydration response via Gardos channels proved to be a stochastic phenomenon in the RBC population (Lew et al., 1997). The deoxy-induced increase in permeability was named P_sickle_. In cell-attached patch clamp recordings from sickle RBCs (Vandorpe et al., 2010), deoxygenation-induced P_sickle_ was found to be inhibited by GsMTx4, a specific inhibitor of mechanosensitive channels (Ostrow et al., 2003; Bae et al., 2011). In normal RBCs, the release of ATP under physiological levels of shear was shown to depend on the presence of external calcium and was also inhibited by GsMTx4, supporting the view that PIEZO1 mediates Ca^2+^ influx under normal circulatory shear stress (Larsen et al., 1981; Cinar et al., 2015).

Based on these considerations, PIEZO1 appears as a prime candidate for the mediation of RBC densification in normal RBC senescence, and also as the channel responsible for P_sickle_, a key participant in the mechanism of sickle cell dehydration (Lew and Bookchin, 2005). It is the mechanical gating of this channel that makes PIEZO1 such a good candidate for translating stretch-induced circulatory RBC deformations into micro-dehydration events in normal senescence, and for polymer-induced membrane protrusions into P_sickle_ in sickle cells.

How would PIEZO1 lead to the dehydration of senescent RBCs? Because of its poor ion selectivity (Gnanasambandam et al., 2015), PIEZO1 activation would in principle contribute to general ion gradient dissipation. While minimal for Na^+^, K^+^, and Mg^2+^ with each brief PIEZO1 opening, the steep inward electrochemical gradient of Ca^2+^ would be expected to generate a transient peak of elevated [Ca^2+^]_i_. A significant proportion of such peaks may reach [Ca^2+^]_i_ levels activatory to Gardos channels, as with stochastic P_sickle_, with cumulative downstream effects of selective KCl loss in excess of NaCl gains, leading to progressive and generalized cell dehydration and densification.

Participation of Gardos channels in the age-dependent densification of human RBCs has been suggested many times in the past. The required [Ca^2+^]_i_ elevations were attributed mostly to progressive PMCA weakness, aided by increases in Ca^2+^ permeability through undefined pathways. Given the large spare capacity of the PMCA it is difficult to estimate its real contribution. A weakening calcium pump may increase the frequency with which PIEZO1 generates Ca^2+^ peaks high enough to activate Gardos-channels, and also extend the duration of their active state by delayed Ca^2+^ extrusion (Dyrda et al., 2010).

## On the mechanism of programmed senescence

The picture emerging from these considerations outlines a sequence of transport and homeostatic changes aimed at preserving the volume of aging RBCs within a narrow OVR range for as long as energy from fading metabolism, weakening sodium pumps, and enfeebled cation gradients can be sustained. An outline consistent with current knowledge suggests that the swelling tendency resulting from the declining activity of the sodium pump is opposed by net KCl and fluid losses resulting from periodic Gardos channel activation during capillary passages, elicited by brief surges in cell calcium via stretch-activated PIEZO1channels. The long-term cumulative effects of these opposing transport-mediated processes are a progressive dissipation of the sodium and potassium gradients, and a balanced volume control with a marginal, not functionally compromising increase in cell density for most of the lifespan of the cells. At some advanced stage, variable in RBCs from different subjects, sodium pump weakness and potassium gradient dissipation reach levels that can no longer prevent sustained net NaCl gains and RBC rehydration. This causes the densification trend to reverse and RBCs to swell, somehow signalling for terminal removal along the way (Kay, 1975; Beutler, 1986; Lutz and Bogdanova, 2013). In this light, terminal density reversal appears as a way of prolonging RBC longevity in a fit functional state within the OVR range, an opportunistic lifespan extension enabled by the preceding densification period. Thus, RBCs appear planned by evolution to last for as long as fit to function, not for obsolescence (Beutler, 1986).

This narrative outlines a hypothesis for the evolution and mechanism of the extended longevity of human RBCs consistent with current knowledge. At proof stage, a paper was published providing powerful new evidence in support of the mechanism hypothesized here. Kuchel and Shishmarev (2017) combined nuclear magnetic resonance spectroscopy measurements of ^13^C signals of lactate production and of ^133^Cs signals of K^+^ congener fluxes with a most elegant experimental design in which RBCs embedded in gelatin gels of varied compositions could be exposed to reversible deformation protocols. Their results showed a clear calcium-dependent link between mechanical deformations, increased lactate production and increased Cs(K) fluxes. Additional experiments with the PIEZO1 activator yoda1 and PIEZO1 inhibitor GsMTx4 unambiguously identified PIEZO1 as the mediator of the deformation effects, starting with PIEZO1 activation allowing down-gradient Ca^2+^ influx. The ensuing [Ca^2+^]_i_ elevation stimulates PMCA activity with downstream lactate production, and activates Gardos channels increasing net Cs(K)-salt efflux and fluid loss. This is the same sequence suggested to participate in myriad micro-quantal deformation events in the capillary circulation as part of the hypothesized OVR control mechanism of RBC longevity.

## Author contributions

All authors listed have made a substantial, direct and intellectual contribution to the work, and approved it for publication.

### Conflict of interest statement

The authors declare that the research was conducted in the absence of any commercial or financial relationships that could be construed as a potential conflict of interest.
